# Supplementary material for: Highly Accurate Estimation of Cell Type Abundance in Bulk Tissues Based on Single‐Cell Reference and Domain Adaptive Matching
Source: Adv Sci (Weinh). 2023 Dec 10;11(7):2306329. doi: 10.1002/advs.202306329 (PMC10870031; doi:10.1002/advs.202306329)
Supplement: Supplementary file 1 — Supporting Table 1 [file ADVS-11-2306329-s002.pdf]

ADVANCED SCIENCE
Open Access

Supporting Information

for *Adv. Sci.*, DOI 10.1002/advs.202306329

Highly Accurate Estimation of Cell Type Abundance in Bulk Tissues Based on Single-Cell Reference and Domain Adaptive Matching

Xinyang Guo, Zhaoyang Huang, Fen Ju, Chenguang Zhao and Liang Yu**

(A) Smart-seq2 reference and 10x Chromium pseudobulk

	SCROAM	Bisque	MuSiC	NNLS	SCDC
Kidney	0.080	0.093	**0.042**	0.043	0.252
Large intestine	**0.066**	0.150	0.081	0.087	0.177
Liver	0.121	0.081	0.056	0.071	**0.054**
Lung	**0.066**	0.100	0.074	0.075	0.068
Pancreas	0.113	0.101	**0.100**	0.110	0.158
Skin	**0.051**	0.281	0.099	0.101	0.413
Thymus	**0.070**	0.139	0.151	0.247	0.172
Trachea	**0.074**	0.081	0.077	0.121	0.130
Mean	**0.08**	0.128	0.085	0.107	0.178

(B) 10x Chromium reference and Smart-seq2 pseudobulk

	SCROAM	Bisque	MuSiC	NNLS	SCDC
Kidney	**0.046**	0.075	0.050	0.060	0.074
Large intestine	0.154	0.150	**0.105**	0.112	0.160
Liver	0.046	0.057	**0.032**	0.052	0.056
Lung	**0.067**	0.093	0.071	0.068	0.070
Pancreas	0.107	**0.070**	0.103	0.115	0.099
Skin	**0.053**	0.281	0.067	0.061	0.119
Thymus	**0.165**	0.177	0.217	0.265	0.267
Trachea	0.152	**0.087**	0.149	0.185	0.178
Mean	**0.099**	0.124	0.099	0.118	0.128

Table S1: Deconvolution errors of different methods in the pseudo-bulk experiments. Smart-seq2 and 10x Chromium data from the Tabula Muris Senis experiment were used, and the specified methods were used for deconvolution in eight organs. The presented values are the L1 distance between the true and inferred values, divided by the number of cell types. These values correspond to Figure 2 in the main text.
