# Supplementary material for: Highly Accurate Estimation of Cell Type Abundance in Bulk Tissues Based on Single‐Cell Reference and Domain Adaptive Matching
Source: Adv Sci (Weinh). 2023 Dec 10;11(7):2306329. doi: 10.1002/advs.202306329 (PMC10870031; doi:10.1002/advs.202306329)
Supplement: Supplementary file 2 — Supporting Table 2 [file ADVS-11-2306329-s003.pdf]

ADVANCED SCIENCE
Open Access

Supporting Information

for *Adv. Sci.*, DOI 10.1002/advs.202306329

Highly Accurate Estimation of Cell Type Abundance in Bulk Tissues Based on Single-Cell Reference and Domain Adaptive Matching

Xinyang Guo, Zhaoyang Huang, Fen Ju, Chenguang Zhao and Liang Yu**

Method	Estimated proportions(%) 60% MDA-MB468	30% MCF-7	10% Fibroblasts	Mean L1 error
NNLS	22	56	21	25
MuSiC	65	24	10	3.7
Bisque	66	22	11	3.7
SCDC	64	26	11	3
SCROAM	60	34	6	**2.7**

Table S2: Results on the cell line mixture dataset. The data with known cell type proportions from Dong et al. (2021) were used to evaluate each applicable method (displayed proportions may not sum up to 1 due to rounding). These values correspond to Figure 4 in the main text.
