# Supplementary material for: Highly Accurate Estimation of Cell Type Abundance in Bulk Tissues Based on Single‐Cell Reference and Domain Adaptive Matching
Source: Adv Sci (Weinh). 2023 Dec 10;11(7):2306329. doi: 10.1002/advs.202306329 (PMC10870031; doi:10.1002/advs.202306329)
Supplement: Supplementary file 3 — Supporting Table 3 [file ADVS-11-2306329-s001.pdf]

ADVANCED SCIENCE
Open Access

# Supporting Information

for *Adv. Sci.*, DOI 10.1002/advs.202306329

Highly Accurate Estimation of Cell Type Abundance in Bulk Tissues Based on Single-Cell Reference and Domain Adaptive Matching

*Xinyang Guo, Zhaoyang Huang, Fen Ju, Chenguang Zhao* and Liang Yu**

| Dataset name | Description | sc/bulk RNA-seq | Accession ID |
| --- | --- | --- | --- |
| Tabula Muris Senis | Many mouse organs | Both | GSE13204 |
| Dong et al. (2020) | Human fibroblasts, cell lines | Both | GSE136148 |
| Newman et al. (2019) | Human PBMCs | scRNA-seq | GSE127417 |
| Newman et al. (2019) | Human PBMCs | bulk RNA-seq | GSE127813 |
| 10x Genomics data sets | Human PBMCs | scRNA-seq | https://support.10xgenomics.com/single-cell-gene-expression/datasets. |
| Monaco et al. (2019) | Human PBMCs | bulk RNA-seq | GSE107011 |
| Xie et al. (2020) | Human neutrophils | scRNA-seq | GSE137540 |
| | | | |
| scGBM | Glioblastoma | scRNA-seq | GSE103224 |
| TCGA-GBM | Glioblastoma | bulk RNA-seq | https://portal.gdc.cancer.gov |
| Normal Liver | Normal Liver | scRNA-seq | GSE115469 |
| TME-Stroma | Tumor microenvironment-Stroma | scRNA-seq | GSE146409 |
| TCGA-LIHC | Fibrolamellar Carcinoma(3)/ HCC(427)/ Hepatocholangiocarcinoma (8) | bulk RNA-seq | https://portal.gdc.cancer.gov |

Table S3: Description of the datasets used. Dataset name - the name of the dataset; Description - the species and organ/tissue profiled; sc/bulk RNA-seq - the protocol used to detect expression; Accession ID - the location number where the dataset can be found.
